# Pathovar Transcriptomes

**DOI:** 10.1128/mSystems.00049-17

**Published:** 2017-07-25

**Authors:** Amy Platenkamp, Jay L. Mellies

**Affiliations:** Biology Department, Reed College, Portland, Oregon, USA

**Keywords:** diversity, EPEC, pathovar, transcriptome, genome analysis

## Abstract

Archetypal pathogenic bacterial strains are often used to elucidate regulatory networks of an entire pathovar, which encompasses multiple lineages and phylogroups. With enteropathogenic *Escherichia coli* (EPEC) as a model system, Hazen and colleagues (mSystems 6:e00024-17, 2017, https://doi.org/10.1128/mSystems.00024-17) used 9 isolates representing 8 lineages and 3 phylogroups to find that isolates with similar genomic sequences exhibit similarities in global transcriptomes under conditions of growth in medium that induces virulence gene expression. They also found variation among individual isolates. Their work illustrates the importance of moving beyond observing regulatory phenomena of a limited number of regulons in a few archetypal strains, with the possibility of correlating clinical symptoms to key transcriptional pathways across lineages and phylogroups.

## Abstract (precis)

Archetypal pathogenic bacterial strains are often used to elucidate regulatory networks of an entire pathovar, which encompasses multiple lineages and phylogroups. With enteropathogenic *Escherichia coli* (EPEC) as a model system, Hazen and colleagues (mSystems 6:e00024-17, 2017, https://doi.org/10.1128/mSystems.00024-17) used 9 isolates representing 8 lineages and 3 phylogroups to find that isolates with similar genomic sequences exhibit similarities in global transcriptomes under conditions of growth in medium that induces virulence gene expression, and they found variation among individual isolates.

## COMMENTARY

Hazen et al. examined whether global transcriptome similarity can be correlated to genetic relatedness of phylogenetic lineages of an *Escherichia coli* diarrheal pathovar (a set of strains with similar characteristics) of global significance (1). Previously, the group had used RNA sequencing (RNA-Seq) analysis to understand the transcriptomes of four prototypical enteropathogenic *E. coli* (EPEC) strains in different media and phases of growth, as well as under conditions of adherence to epithelial cells in culture. Under conditions mimicking those associated with pathogenesis, they observed coordinate regulation of key metabolic and virulence genes (2). Thus, the group has sought to gain a far greater understanding of transcriptional control of virulence by EPEC. In their most recent work, Hazen et al. analyzed the transcriptomes of 8 typical EPEC isolates containing the signature virulence plasmid pEAF and one atypical isolate lacking the plasmid, representing different lineages (1). Differing genetic backgrounds in typical and atypical EPEC isolates have previously been shown to impart alternate disease outcomes—typical EPEC causes profuse watery diarrhea in children, while atypical EPEC lacking the bundle-forming pilus carried on the pEAF plasmid is associated with persistent disease (3, 4). Here they show that genomic sequence analysis in combination with expression data obtained via RNA-Seq analysis permits researchers to move beyond solely studying prototypical or archetypal strains.

Hazen and colleagues concluded that the number of genetically similar lineages that have similar global transcriptomes is greater than the number of dissimilar lineages that have similar global transcriptomes. Their report also illustrates the subtleties of global transcriptomes across different isolates and lineages, and perhaps that represents the more significant observation. The diversity that they observed was due to genetic variation but also to variation in the regulatory control of key virulence genes and to subtleties and contradictions, in contrast to results shaped by limitations of technology or approach. The regulatory variation is likely adaptive for the pathogen. To illustrate this point, only 21 conserved core genes were significantly differentially expressed in all 9 of the EPEC isolates under virulence-inducing versus noninducing conditions. The list included metabolic genes but lacked most of the virulence genes thought to be regulated under these conditions. Through genomics and transcriptomics, we will gain a better understanding of the consistencies and inconsistencies across the different EPEC lineages and, ultimately, a clearer picture of the regulatory networks necessary to cause disease. Their work highlights that an integrative and inclusive approach to improve our understanding of the greater pathovar is not only possible but imperative.

The E2348/69 strain of typical EPEC, the first *E. coli* pathovar to be described, was isolated in 1969 and has been studied as a prototypical strain over several decades, as a model pathogen causing diarrhea in children. This work by Hazen et al. reminds us of the historical and practical importance of studying prototypical isolates, clearly illustrates the limitations of this approach, and opens a new door to a much greater understanding of transcriptional control of bacterial disease. Many researchers around the globe study EPEC pathogenesis, including those investigating virulence gene expression, but all the isolates studied most likely contain genetic and regulatory variation compared to the original isolate due to genetic differences and laboratory passage over time (5). Furthermore, the necessity of being able to compare data reported by researchers focusing on a few prototypical strains, even under consistent growth conditions, limits overall understanding of regulatory phenomena.

As pointed out by the authors, we can begin to investigate whether clinical symptoms can be correlated with differences in transcriptional regulation and to understand why some lineages might be bystanders as opposed to true pathogens and why some lineages are associated with more-severe disease than others. With genome sequencing providing information about gene content and single nucleotide polymorphisms and RNA-Seq analysis routinely available to understand transcriptomes, some researchers have suggested that we can now move beyond the pathovar distinction for diagnostic purposes (6). If their prediction is correct, clinical symptoms can be correlated to differential transcription of key metabolic and virulence genes. The work by Hazen and colleagues offers the beginning of a much more nuanced and yet comprehensive understanding of how different *E. coli* lineages are transmitted and cause disease outbreaks.
